# Pityriasis Rubra Pilaris in an Atopic Dermatitis Patient: A Case Report

**DOI:** 10.7759/cureus.67077

**Published:** 2024-08-17

**Authors:** Mehad Almoqati, Lama Almasoudi, Zainab Alfaqih, Sarah M Fageeh, Khalid Al Hawsawi

**Affiliations:** 1 College of Medicine, Taif University, Taif, SAU; 2 Faculty of Medicine, Umm Al-Qura University, Makkah, SAU; 3 Dermatology, King Abdulaziz Hospital, Makkah, SAU

**Keywords:** atopic dermatitis, nbuvb, ppk, type ii prp, prp, atypical adult pityriasis rubra pilaris

## Abstract

Pityriasis rubra pilaris (PRP) is a rare, chronic, inflammatory papulosquamous skin disease. Here, we report a case of a 43-year-old female with a known history of atopic dermatitis, bronchiectasis, and goiter who presented with a six-month history of persistent itchy skin lesions on her extremities. A skin examination revealed multiple diffuse, well-defined, fine, scaly erythematous patches with areas of spared skin over all four extremities, along with palmoplantar keratoderma. The trunk was spared. The differential diagnosis included atopic eczema, pityriasis rubra pilaris, dermatomyositis, mycosis fungoides, parapsoriasis, psoriasis, and drug-induced eczematous dermatitis. A skin biopsy revealed hyperkeratosis, acanthosis, spongiosis, follicular plugging, and mild perivascular lymphohistiocytic cellular infiltrates in the dermis. Based on the clinicopathological findings, the patient was diagnosed with atypical adult pityriasis rubra pilaris (PRP) (type II). She was started on narrowband UVB phototherapy (NBUVB). Two months after starting NBUVB treatment, all the skin lesions had cleared. She was put under periodic follow-up, and the lesions have remained clear for two years up to the time of this publication.

## Introduction

Pityriasis rubra pilaris (PRP) is an uncommon inflammatory papulosquamous condition affecting both adults and children. Six distinct types of PRP have been identified based on clinical characteristics, age of onset, and prognosis. The etiology is unknown. The disease’s pathophysiology includes impaired immune function, poor keratinization, abnormal vitamin A metabolism, and CARD14 mutations. Triggering factors include infection, ultraviolet exposure, minor traumas, and drugs [1-5]. PRP is characterized by erythematous scaly patches with areas of spared skin, follicular hyperkeratosis on an erythematous base, and yellowish palmoplantar keratoderma (PPK) [6]. The lesions usually start on the head and neck and spread downward to the body [7]. Type II PRP, also known as atypical adult PRP, affects 5% of all PRP cases [8]. It is characterized by ichthyosiform scales, PPK with coarse lamellated scales, and occasionally alopecia [9]. Here, we report a case of a 43-year-old female with a known history of atopic dermatitis, bronchiectasis, and goiter, who was diagnosed with atypical adult PRP (type II).

## Case presentation

**Figure 1:**
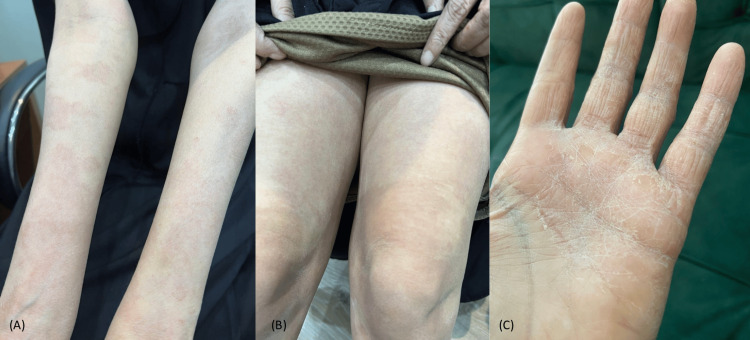
Multiple, diffuse, well-defined, fine, scaly erythematous patches with an area of spared skin on (A) upper extremities, (B) lower extremities, and (C) palmoplantar keratoderma

**Figure 2:**
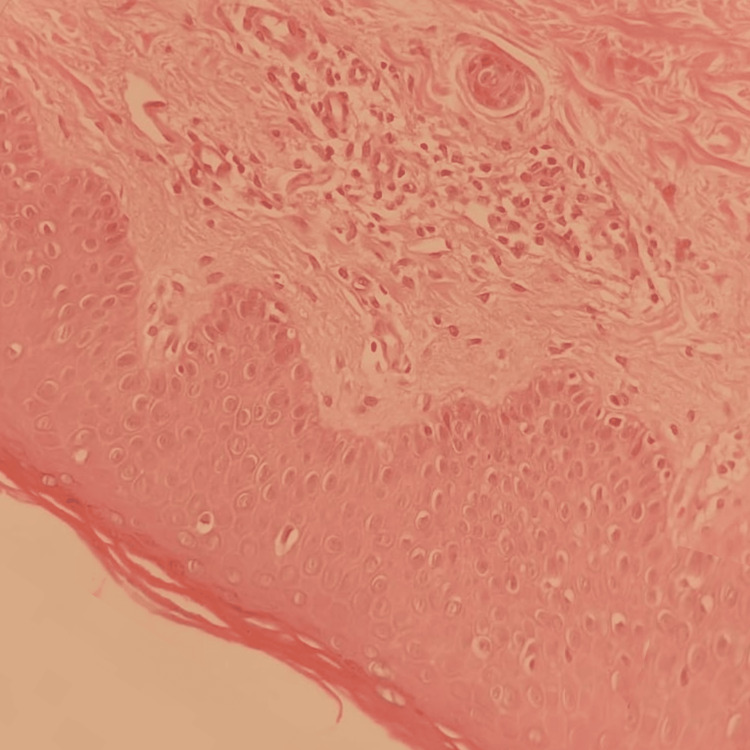
Mild hyperkeratosis, acanthosis, spongiosis, follicular plugging, and mild perivascular lymphohistiocytic cellular infiltrates in the dermis

**Figure 3:**
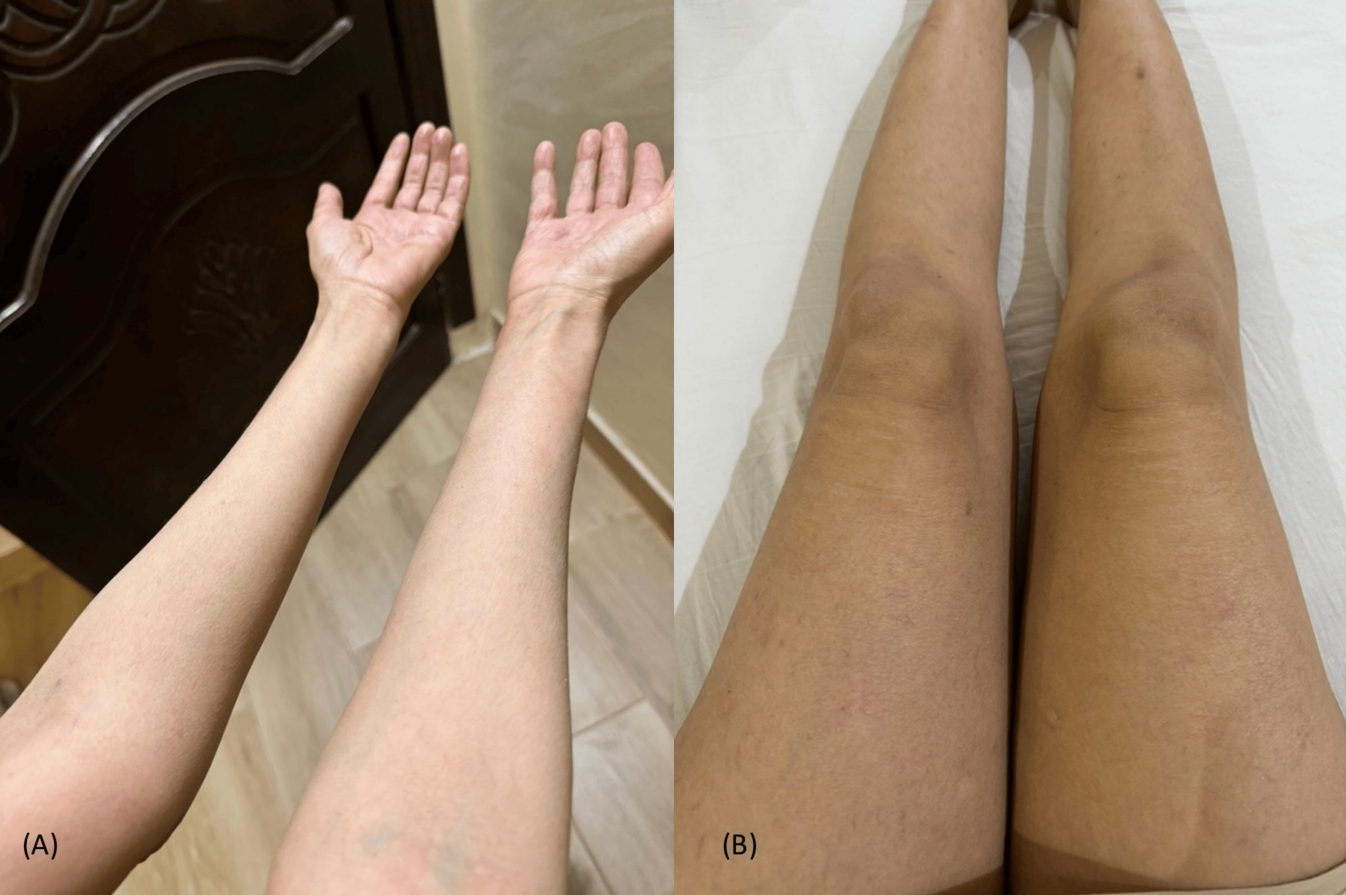
Complete resolution after two months of starting NBUVB therapy: (A) upper extremities, (B) lower extremities NBUVB: narrowband ultraviolet B phototherapy

A 43-year-old female presented to our clinic with a 6-month history of persistent itchy skin lesions on her extremities. The skin lesions started on the upper extremities, including the palms, and then spread down to the lower extremities, including the soles. She had mild atopic dermatitis on her upper extremities that was well-managed with a topical steroid. Unlike her chronic atopic dermatitis, the new rash did not respond to treatment with medium potency topical steroid (MPTS) as betamethasone valerate. Moreover, the patient had been diagnosed with bronchiectasis and was on albuterol and periodic IV antibiotics. She also had a goiter, which was under observation without active intervention. There were no similar cases in her family. A skin examination showed multiple, well-demarcated, fine, scaly erythematous patches with areas of spared skin on all four extremities associated with palmoplantar keratoderma (Figure 1). The differential diagnosis included atopic eczema, pityriasis rubra pilaris, dermatomyositis, mycosis fungoides, parapsoriasis, psoriasis, and drug-induced eczematous dermatitis. A skin biopsy was taken, which revealed mild hyperkeratosis, acanthosis, spongiosis, follicular plugging, and mild perivascular lymphohistiocytic cellular infiltrates in the dermis (Figure 2). The laboratory workup was normal for complete blood count (CBC), muscle enzymes, liver enzymes, serum urea, and creatinine. Based on the above clinicopathological findings, the patient was diagnosed with atypical adult pityriasis rubra pilaris (PRP) (type II). She was started on narrowband ultraviolet B phototherapy (NBUVB). Following two months of NBUVB treatment, complete resolution of all skin lesions was observed (Figure 3). Subsequently, the patient was subjected to periodic follow-up, and the lesions have remained in remission for two years up to the time of this publication.

## Discussion

PRP is a rare, inflammatory, papulosquamous dermatological disorder [1]. The spectrum of PRP variants is categorized into six distinct subtypes, including classic adult PRP, atypical adult PRP, classic juvenile PRP, circumscribed juvenile PRP, atypical juvenile PRP, and human immunodeficiency PRP [2]. A PRP diagnosis can be difficult to make due to the presence of similar conditions, including atopic eczema, dermatomyositis (DM), mycosis fungoides (MF), parapsoriasis, psoriasis, and drug-induced eczematous dermatitis [10]. Although the skin biopsy results in this case were nonspecific, as can be seen in atopic dermatitis and partially treated PRP as seen in our case, as she received topical corticosteroid. However, the well-demarcated skin lesion borders seen in our patient are against atopic dermatitis. Also, there were no features suggestive of DM (Wong type), MF, or psoriasis. The atypical acral localization of the skin lesions added more complexity to the diagnosis of PRP. The typical persistent, fine, scaly, well-defined erythematous patches with islands of normal skin and the ichthyosiform scales and the patient’s rapid response to NBUVB therapy suggested a diagnosis of PRP. Massa A et al. present a case of an eight-year-old girl with mixed type III/IV PRP who showed 90% improvement after two months of initiating NBUVB treatment [11]. Another study reported treatments for PRP, including systemic isotretinoin, acitretin, phototherapy, and systemic immunosuppressants either alone or in combination [12].

## Conclusions

Our case underscores the diagnostic challenges of PRP, especially with its atypical presentations and overlapping characteristics with other dermatological conditions such as atopic eczema. Despite nonspecific biopsy results, the persistent scaly erythematous patches with islands of normal skin and ichthyosiform scales, and rapid improvement with NBUVB therapy pointed toward PRP. This case emphasizes the need to consider PRP in the differential diagnosis of papulosquamous disorders to ensure effective management and improved patient outcomes.
